# A Review of Antiseptic Wound Treatment

**Published:** 1886-05

**Authors:** A. C. Walker

**Affiliations:** Rockdale, Texas


Medical ★ Journal,

PUBLISHED MONTHLY AT

AUSTIN, TEXAS.

Vol. I.] MAY, 1886. [No. 11.

*"Scribimus indocti, doctique!"*

### Original Articles.

CONTRIBUTED EXCLUSIVELY TO THIS JOURNAL.

*The Articles in this Department are accepted, and published with the understanding that we are not responsible for, nor do we indorse the views and opinions of the writers, by so doing.*

### A REVIEW OF ANTISEPTIC WOUND TREATMENT.

*By A. C. Walker, M. D., Rockdale Texas.*

Read before Milam County Medical Society, and voted to be printed in (Daniel's TEXAS MEDICAL JOURNAL.)

IT has been said by an eminent writer, that the treatment of wounds is undoubtedly not merely the first stone, but the corner stone of surgery; and while I am confident that every member here is perfectly familiar with the aseptic treatment of wounds, and antiseptic surgical dressings, still I feel the assurance that all who have the true interest of our profession at heart, will gladly enter into the spirit of a short review on the subject of antiseptic dressings.

This discovery or advancement in the treatment of wounds, has created a new era in the science of surgery, and opened up a field of research and investigation which has already reaped rich harvests of brilliant success in the prophylaxis of wound infection, lessening the duration of confinement from injures, and the absolute saving of human life and preservation of limbs. Only a few years ago, while a student at Bellevue, I remember well that the mortality following certain grave surgical operations was extremely discouraging to surgeons. At that time and for years afterwards, at that distinguished hospital it was a matter of record that recovery from amputations of the thigh was the exception. Resections of the larger joints were rarely made with favorable results, owing to the excessive suppuration following the operation, with septicæmic complications, hectic fevers and exhaustive drain and shock upon the entire system; and if the patient was fortunate enough to recover at all, it was after a battle of life and death for months and months.

Fractures of a compound nature were looked upon with serious import; and if at all complicated were thought to be hopeless for limb safety, and often limbs were sacrificd which could easily have been saved,as has since been demonstrated, by the present method of treatment. Traumatic injuries of all characters, simple and compound, were every ready to take on different phases of virulent inflammation, either in the form of erysipelas, cellulitis or gangrenous sloughing; and after an amputation a limited degree of sloughing of the flaps was a matter of no surprise or concern.

Secondary hemorrhages were of frequent occurrence; and, when we think of the extensive ulcerated surfaces, exposed, by the old system, the enormous quantity of offensive and putrid discharges poured out, and often perfect sloughs of necrosed tissue thrown off, it is a wonder such accidents were not met with oftener than they were. On account of the discharges of the suppurating surfaces it was necessary to change the dressings of the wounds every day, or at farthest every other day; and by these frequent disturbances of the part, the patient was necessarially subjected to pain, and nervous excitement, but most important the granulating surfaces and surrounding tissues were deprived of *rest*, the most requisite condition for the fruitful union of a wound.

As far back as the time of John Bell, the subject of primary union of wounds was discussed at much length, and by some it was claimed that primary union did occasionally occur under favorable conditions but; the eminent Surgeon Bell "hooted" at the idea, and said "these tales are told with more confidence than veracity, and that immediate coalesence without suppuration was opposed to the rules of nature." Thus illustrating a principle in surgery that had existed from the earliest days of surgery, and so deeply engrafted into the minds and teachings of surgeons that nothing but a revulsion so glaring in its results could have proselyted the profession to the unquestionable verity of antisepticism.

Prior to the writings of Prof. Lister in 1865 on the "antiseptic method" there was nothing decided or harmonious in the theory and principles of wound treatment.

By scientists two views were held regarding the occurrence of fermentation. One class believed that the fermentation and putrifactive changes were due to the gases of the air, oxygen contributing the most potent factor; another class thought that such changes were generated from the influences of organic fluids after exit from tissues and vessels of the body.

With the existence of these theories of putrid fermentation, rigid efforts were made to exclude these noxious vapors from wound surfaces; the air and gases were shut out from wounds by hermetically sealing them with such agents as collodion or styptic colloid, but still the putrifactive process continued regardless of the exclusion of the supposed infecting material. About this time it was found that by the addition of some of the balsams and essential oils, putrifaction was greatly retarded, and unpleasantness of odor was prevented, and these agents were called *antiseptics*.

However, little was done to utilize these valuable agents at that time, only in a very crude and desultory manner, until the publication in 1859 of a paper of Corne & Demeaux on the treatment of wounds by coal tar, from which, for several years very fruitful results were obtained.

Lamarre, a distinguished French surgeon, becoming interested in the success of the *coal tar treatment*, investigated the method more closely, and ascertained that *carbolic acid* was the chief antiseptic constituent in coal tar, and actively introduced it into surgical practice.

Prof. Lister in 1865 awakened to the importance and the success of the carbolic acid treatment, and stimulated by the investigations of Pasteur's results on spontaneous generation and the causes of fermentation, directed his attention to the antiseptic method of treatment. He soon published his views on, and experiments with, carbolic acid, with a fine report of his wonderful surgical success by this method, the result of which has made him the hero of antisepsis, and his name immortal in surgery.

The advancing popularity of the germ theory and the investigations of Schwann, Schroeder, and Pasteur, developed the fact that the theory that fermentation was due to the gases of the air had become untenable. Experiments were made with urine by boiling and allowing the access of only such air as had been passed through a heated tube, and it was found that the fluid could be preserved indefinitely without any evidence of fermentive changes.

Therefore it was inferred that fermentation must be due to some material floating in the air, and capable of destruction and removal by mechanical and chemical means.

It is essential from a surgical standpoint to know that suppurative and purtrifactive changes are strictly a process of fermentation, the causes of which are *particles* which reach the fermentescible substance from the outer world; and these micro-organisms have been called Scizomycettes or Bacteria. These micro-organisms are found in the air, water, and more especially in particles of dust. These bodies have life; they must breathe, take in material for their sustenance, renew their protoplasm and excrete their waste products.

These particles are always present in fermenting fluids, and it would be impossible to obtain fermentation without some form of micro-organism; and it may be stated as a principle, that fermentation is the result of the growth of micro-organisms in fermentescible materials.

The species of micro-organisms that have been identified as capable of producing disease in the human body, are comparatively few in number, and of these the ones that are concerned in the production of the inflammations, and the infection-diseases that complicate wounds, are embraced in two great groups, viz.: Spherical organisms or Micrococci; and rod-shaped organisms, which include Bacteria and Baccilli. It becoming an established fact, that wound suppuration was undoubtedly the result of the fermentive action of living germs infesting the atmosphere, and in abundance deposited upon external objects, and demonstrated by actual experiment that Carbolic-acid possesses the power of sterilizing and even producing the actual death of these organisms, Prof. Lister adopted this method, which has been called Listerism, or antisepticism. After the use by surgeons of Lister's method for several years, it was observed, that failure in wound treatment was of no uncommon occurance, and the faith in Listerism was somewhat shaken; but the more zealous in the study of antiseptics were sure that the principle was correct, but the agent employed, carbolic-acid, was not the germicide that it was reputed to be; and upon closer study and observation it was found, that in order to destroy the septic germs or Bacteria, the strength of the carbolic-acid used, must necessarily be such as to seriously irritate, if not destroy the wounded tissues. In endeavoring to secure an agent as efficient in its work as carbolic-acid, but less objectionable on other accounts, it was found that corrosive sublimate and iodoform were the antiseptics *par excellence.*

In order to establish an aseptic condition of a wound, a number of points must be attended to; the principle of which, involved in carrying out antisepsis, will embrace two methods:

First, those that are required to prevent the accumulation, and to ensure the removal of whatever substance might afford a pabulum favorable to the growth and increase of septic organisms: To facilitate the removal of septic products if formed: And to prevent the introduction into the wound of any substance capable of inducing septic changes in it; these methods are embraced in the single idea, *cleanliness.*

Second, the employment of substances as applications to wound surfaces, which are antagonistic to septic organisms, destroying them or restraining their growth—*antiseptics* in the strict sense of the term.

Under the head of cleanliness stands most prominently the prevention of the introduction into the wound surfaces of all forms of micro-organisms that abound so plentifully on the *skin* ; different varieties of organisms infesting different localities; therefore adjacent tissues to a wound should be scrupulously cleansed and purified. Drainage too, may come under the head of cleanliness, as retention of secretion of exudate serum so abundantly poured out upon the cut surface, after the infliction of a wound, forms a pabulum rich in properties for the culture and growth of septic germs.

Hæmostasis too may be classed under the head of cleanliness; while blood of itself, in the form of a crust, may exclude the introduction of septic material; but it is only so under exceptional conditions. A blood clot located between the surfaces of a wound, is unable to undergo absorption or organization, therefore it can but act as a foreign material and foil all efforts at primary union. The cleanliness of extraneous objects is of prime importance. The hands of the surgeon and of his asistants, the instruments employed, sponges, ligatures and dressings, all should be rigidly examined, and thoroughly cleansed.

The antiseptic agents employed to sterilize or destroy the vital activity of septic germs, are effectively used by washing off the wounded surfaces, irrigating the wound cavities, and by the employment of dressings so impregnated with antiseptic material that the entrance from without, of germs to wound surfaces, capable of generating a suppurative fermentation, is unlooked for.

I will not attempt to enumerate the countless antiseptic agents that have been suggested and used in wound-treatment, but confine myself to those that are most popular, and at the ready command of every physician and surgeon. The application of the aseptic method to operations, and the dressings of wounds either inflicted by the surgeon, or from accidental origin in detail, is as follows :

1. All instruments to be used, should be immersed in solution of carbolic-acid, 1 to 20.

2. Towels to be used, should also be soaked in either a solution of carbolic-acid 1 to 20, or solution bichloride of mercury, 1 to 2000.

3. Sponges which have been previously rendered antiseptic, washed in solution carbolic-acid, 1 to 40.

4. Catgut ligatures in solution of carbolized oil, or oil of juniper, or absolute alcohol.

5. Drainage-tubes in solution of carbolic-acid, 1 to 20.

6. Basins containing solution of carbolic-acid, 1 to 40, and solution of bichloride, 1 to 2000.

DRESSINGS TO BE USED.

1. Iodoform Gause.
2. Bichloride Gause.
3. Iodoform, pepper-box or blower.
4. Sublimate or Boracic-acid absorbent cotton.
5. Rubber tissue protective.
6. Bandages of different varieties.

To illustrate the different steps of procedure in the antiseptic method, we will take as a case in point, an amputation of the thigh, lower third. The patient anaesthetized, is ready for operation. After an Esmarch's bandage is firmly applied and tourniquet adjusted, the region near the site of amputation is first thoroughly washed or scubbed with soap or warm water, then bathed off with sulph: ether which removes all sebaceous substances that may be left from the washing. Then a third washing with solution of bichloride of mercury 1 to 2000, which makes the surface strictly aseptic.

Towels soaked in sublimate solution and enveloped around the limb, both above and below the site of the incisions.

The surgeon and assistants render themselves antiseptic, by first washing their hands with soap and water, and afterwards in a solution of sublimate.

The instruments used, are taken from a tray in which they have been immersed in a solution of carbolic-acid, 1 to 20; in fact, everything that comes in contact with the wound surface must have been previously antisepticized. The sponges used, are selected from a vessel where they have been immersed in antiseptic fluid, and great care is exercised in the perfect antisepsis of sponges,—often the most fertile of septics carriers.

The limb being severed, all bleeding vessels are promptly ligatured with antiseptic catgut, the ends cut short near the knot; the size of the animal ligature used regulated by the importance of vessel secured. All redundance of tissue, fat or otherwise, that might fail to unite with its opposing surface, should be removed with scissors.

The coaptation of the edges of integument should be carefully done, so that tissues of like nature should be brought surface to surface, the corneous layer should never be allowed to be rolled in, as it would certainly prevent primary adhesion.

The suturing of the flap should likewise be done with catgnt sutures, the continuous or interrupted suture employed.

Several drainage tubes should be inserted in a situation most dependant and favorable to a free exit of fluids, and these tubes, may be secured with either silver or safety-pin.

The coaptated edges are now dusted with iodoform and a layer of iodoform gause neatly applied; sublimate gause is then loosely applied around the stump, so arranged the pressure as to bring all the surfaces of the interior of the wound as near together as possible.

Over the gause several layers of boric or sublimate absorbent cotton is used; over which a sheet of rubber tissue is adjusted and then firm roller bandages are snugly turned, holding the entire dressing in perfect position and giving absolute rest to the wounded tissues. With a dressing of this excellence a wound may remain undisturbed for ten or twelve days, or until there is complete union of the parts.

Antiseptic material of sufficient potency to successfully prevent and arrest wound infection is within the command of every physician and surgeon; and as a field surgeon once said, the safety of the wounded patient greatly depends upon the intelligent management of his first attendant.

With the grand triumphs of the successes of antiseptic surgery before us, we must with one accord procaim that "while surgery has thus always been a noble art, it may therefore now begin to claim for the first time, with some justice, that it is a noble science."

